# Multi-marker risk-based screening for prostate cancer

**DOI:** 10.1177/09691413221076415

**Published:** 2022-03-07

**Authors:** Nicholas J Wald, Jonathan P Bestwick, Joan K Morris

**Affiliations:** 1 Institute of Health Informatics, 4919 University College London, London, UK; 2 Population Health Research Institute, 4915 St George’s University of London, London, UK; 3 Wolfson Institute of Population Health, 4617 Queen Mary University of London, London, UK

**Keywords:** Prostate cancer, prostate specific antigen, human kallikrein-related peptidase 2, screening

## Abstract

**Objective:**

To determine prostate cancer screening performance using prostate specific antigen (PSA) along with other markers, expressing markers in age-specific multiples of the median (MoM), and age.

**Methods:**

A prospective nested case-control study used stored serum from 571 men who died of, or with history of, prostate cancer (cases), and 2169 matched controls. Total, free and intact PSA, human kallikrein-related peptidase 2 (hK2), and microseminoprotein were measured and converted into MoM values. Screening marker distribution parameters were estimated in cases and controls. Monte Carlo simulation used these in a risk-based algorithm to estimate screening performance (detection rates [DRs] and false-positive rates [FPRs]).

**Results:**

Almost all (99%) cases occurred aged ≥55. Marker values were similar in cases who did and did not die of prostate cancer. Combining age, total PSA and hK2 MoM values (other markers added little or no discrimination) yielded a 1.2% FPR (95% CI 0.2–4.8%) for a 90% DR (59–98%) in men who died of or with a prostate cancer diagnosis within 5 years of blood collection (risk cut-off 1 in 20), two-thirds less than the 4.5% FPR using total PSA alone measured in ng/ml for the same 90% DR (cut-off 3.1 ng/ml). Screening performance over 10 years yielded a 33% (22–46%) FPR for a 90% DR.

**Conclusion:**

Screening performed up to every 5 years from age 55 using the multi-marker risk-based screening algorithm for future prostate cancer achieves a high DR and a much lower FPR than using PSA alone, resulting in reductions in overdiagnosis and overtreatment.

## Introduction

In 2017 there were 10,755 deaths from prostate cancer in England and Wales,^1^ and 30,468 deaths in the USA,^2^ representing the second commonest cause of death from cancer in men after lung cancer. A man's age has a strong influence on the risk of prostate cancer. Prostate specific antigen (PSA) has been found to be a discriminatory screening test for prostate cancer; the pooled result from four cohort studies totalling 49,261 healthy men showed that men aged 60–74 who had a PSA level ≥12 times the normal median level had about a 50% chance of developing clinical prostate cancer in the next three years.^3^ Parkes et al. [1995] converted PSA concentrations into age-specific multiples of the median (MoM) in men who did not present or die of prostate cancer.^3^ Whilst gestational age and centre-specific MoM values are widely used in prenatal screening where their utility over simply using mass units is well recognised, the utility of using PSA MoM values instead of mass units has not been adopted.

A drawback of using PSA to screen for prostate cancer is that it can be raised in the absence of prostate cancer in men who have other conditions or in men who have prostate cancer that is not aggressive. Raised PSA in such men essentially leads to false-positive results. Several task forces have recommended against using PSA as a screening test for prostate cancer.^4^ To help overcome the problem of false-positives, other markers have been proposed, such as free PSA and human kallikrein-related peptidase 2 (hK2), a molecule similar to PSA^5^ but different in its enzymatic activity^6^ and beta-microseminoprotein (MSP).^7^

Age and the mass concentrations of total PSA, free PSA, intact PSA and hK2 (sometimes collectively referred to as the kallikrein or 4 K markers) have been incorporated into an algorithm, which has been proposed to predict significant (Gleeson score ≥7) prostate cancer.^8^ The same panel has been proposed as a way of limiting the need for a biopsy in men with raised PSA levels.^9^ In one study, the ratio of free to total PSA had little or no effect on improving screening performance over PSA alone, suggesting that combining all the markers may not be necessary.^10^ We here examine the value of age plus total PSA, intact PSA, free PSA, hK2 and MSP MoM values in different combinations to determine the screening performance of combining the markers that are found to be informative in a risk-based screening algorithm, and do so according to screening interval. We quantify the extent to which the best combination of markers together with age improves screening performance compared to using PSA alone.

## Methods

We used data from the British United Provident Association (BUPA) cohort study to (i) determine the association between PSA and future prostate cancer over different periods of follow-up (interval between blood sampling and prostate cancer registration or prostate cancer deaths recorded on the death certificate); (ii) determine the screening performance in terms of the detection rate (the proportion of men who will be affected during a specified time interval with a positive result, also known as sensitivity) and the false positive rate (proportion of men who will be unaffected during the same time interval with a positive result); (iii) determine whether the screening performance differs between men who died of prostate cancer and those who died of other causes but with a history of prostate cancer; (iv) examine several markers in addition to total PSA, namely free PSA, intact PSA, hK2 and MSP; (v) determine whether expressing PSA, hK2 and MSP in MoM values in men of the same age tested in the same laboratory is more discriminatory with respect to future prostate cancer than PSA expressed in mass units (ng/ml); and (vi) incorporate age and any of the other markers shown to be independently predictive of prostate cancer in a multivariate screening algorithm model using a person's risk of prostate cancer over a specified period of time as the screening variable. The BUPA cohort study was approved by the BUPA Ethics Committee.

Cases of prostate cancer were ascertained from national death records (to obtain deaths from prostate cancer) and from the national cancer registry (to obtain notifications of prostate cancer). A total of 571 affected men (men with prostate cancer [ICD 8 and ICD 9 codes 185, ICD 10 code C61] on death certificates or cancer registrations) were identified from the BUPA cohort study. Of these, 324 died of prostate cancer (ICD 8 and ICD 9 codes 185, ICD 10 code C61 on death certificates) and 247 died with prostate cancer (above codes not on death certificates). Gleason scores were not available. Each was matched (for age at collection of serum sample to within same 5-year group, duration of storage of the serum sample and hence length of follow-up [calendar year] and number of freeze-thaw cycles) to 4 unaffected men (men who did not die of, or with a history of, prostate cancer). Serum samples for all were retrieved from storage, shipped on dry ice, thawed and 0.3 ml pipetted into sample tubes. All laboratory analyses were conducted blind to prostate cancer status. Details of the biochemical analyses are given in the Supplementary Appendix. For 107 unaffected men there was insufficient sample to perform the assays, leading to a total of 2169 unaffected men. In some of the statistical analyses the number of cases and controls is slightly lower than the total due to missing data.

Screening performance was estimated using the multivariate Gaussian distribution method employed in prenatal screening for Down's syndrome.^11^ A screen-positive result was defined as the risk of developing prostate cancer within a specified time interval greater than or equal to a specified risk cut-off. Estimates of the detection rate and false-positive rate at or greater than specified risk cut-offs for specified durations of follow-up were computed. Details of the statistical methods are given in the Supplementary Appendix. Confidence intervals for the estimates of screening performance were derived by bootstrapping, with 500 dataset replications.

## Results

Table 1 shows relevant characteristics of the men in the dataset, namely the age distribution of the men, the length of follow-up between blood sampling and prostate cancer death or prostate cancer registration, and median marker levels in mass units among affected and unaffected men.

**Table 1.** Median age (interquartile range), follow-up groups and median marker levels (interquartile range) in affected (men who died of, or with a history of, prostate cancer) and unaffected men.

	Affected	Unaffected
N	571	2169
Age at baseline	54 (49–59)	54 (49–59)
Follow up		
0–5 years	12 (2.1%)	46 (2.1%)
6–9 years	34 (6.0%)	132 (6.1%)
10–14 years	75 (13%)	291 (13%)
15–19 years	135 (24%)	508 (23%)
20 + years	314 (55%)	1192 (55%)
Markers (ng/ml)		
Free PSA	0.23 (0.15 to 0.39)	0.18 (0.11 to 0.30)
Total PSA	1.48 (0.84 to 3.01)	0.90 (0.53 to 1.61)
Intact PSA	0.13 (0.08 to 0.23)	0.10 (0.06 to 0.18)
hK2	0.037 (0.023 to 0.060)	0.029 (0.018 to 0.044)
MSP	27.8 (19.4 to 37.1))	27.8 (19.8 to 37.6)

PSA: prostate specific antigen; hK2: human kallikrein-2; MSP: beta-microseminoprotein.

Figure S1 shows the increase in median marker levels in ng/ml with increasing age, in unaffected men together with the regression lines. Total, free and intact PSA concentrations followed log-quadratic increases with age (*p* = 0.001, *p* = 0.003 and *p* = 0.010 respectively); log-quadratic fits were statistically significantly better than log-linear fits. hK2 and MSP log concentrations increased linearly (both *p* < 0.001); log-quadratic fits were not statistically significantly better. The regression equations, which were used to calculate MoM values, are shown in the footnote to Figure S1.

Table 2 shows the median MoM values in men who died of prostate cancer and those who died of other causes but with a history of prostate cancer after 0–5 years of follow-up, 0–10 years of follow-up and at any time. The MoM values decreased with increasing intervals between sample collection and length of follow-up. There were no statistically significant differences in marker values between men who died of prostate cancer and those who died having had a registration of prostate cancer.

**Table 2.** Median marker multiple of the median values among men who died of prostate cancer (PC) and men who died with a history of PC according to length of follow-up.

	PC cause of death		Died with a history of PC but not of PC		
	n	Median (95% CI)	n	Median (95% CI)	*p*-value
Follow up ≤5 years (n = 12)					
Free PSA	8	5.99 (2.62 to 13.69)	4	6.15 (1.54 to 24.53)	0.734
Total PSA	8	14.41 (5.94 to 34.97)	4	7.51 (1.37 to 41.21)	0.396
Intact PSA	8	7.16 (2.88 to 17.79)	4	6.72 (1.72 to 26.18)	0.734
hK2	8	3.19 (2.04 to 5.01)	4	3.20 (0.59 to 17.38)	0.865
MSP	8	0.57 (0.31 to 1.05)	4	0.79 (0.15 to 4.28)	0.734
Follow up <10 years (n = 46)					
Free PSA	30	1.99 (1.32 to 3.00)	16	2.86 (1.71 to 4.81)	0.433
Total PSA	30	4.26 (2.63 to 6.92)	16	4.17 (2.40 to 7.24)	0.854
Intact PSA	30	2.11 (1.39 to 3.18)	16	3.25 (1.84 to 5.73)	0.729
hK2	30	2.13 (1.57 to 2.89)	16	1.59 (0.81 to 3.12)	0.475
MSP	30	0.83 (0.67 to 1.02)	16	1.20 (0.77 to 1.86)	0.189
All data (n = 569)					
Free PSA	322	1.26 (1.14 to 1.40)	247	1.30 (1.17 to 1.44)	0.490
Total PSA	322	1.70 (1.50 to 1.93)	247	1.66 (1.47 to 1.87)	0.937
Intact PSA	320	1.27 (1.15 to 1.41)	246	1.28 (1.14 to 1.43)	0.348
hK2	322	1.28 (1.14 to 1.43)	246	1.25 (1.10 to 1.42)	0.704
MSP	322	1.00 (0.93 to 1.07)	247	1.05 (0.95 to 1.16)	0.159

PSA: prostate specific antigen; hK2: human kallikrein-2; MSP: beta-microseminoprotein.

**Figure 1.**
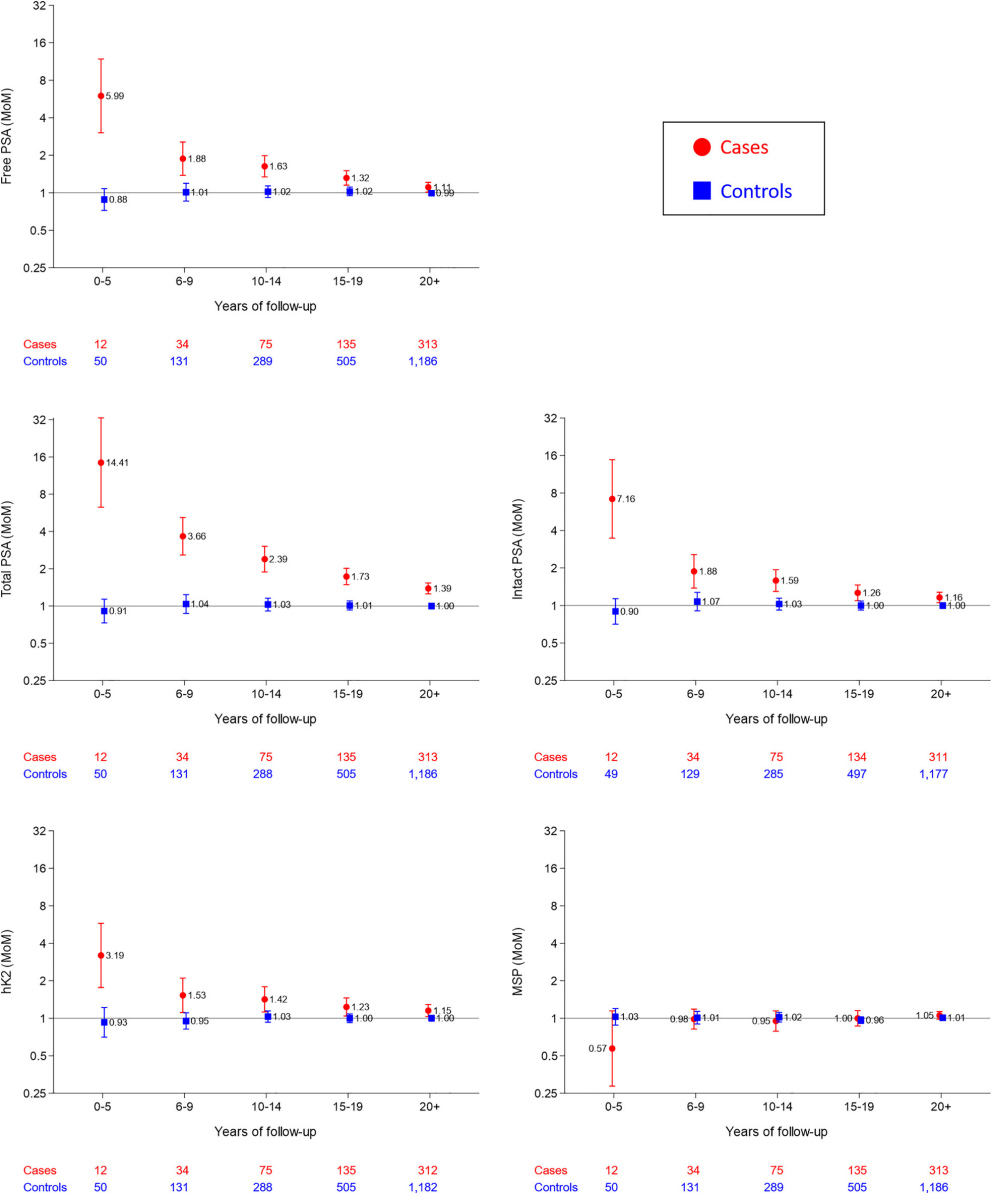
Median marker multiple of the median (MoM) values, with 95% confidence intervals of markers in affected (died of, or with a history or, prostate cancer) and unaffected men according to length of follow-up. PSA: Prostate specific antigen; hK2: Human kallikrein-2; MSP: Beta-microseminoprotein.

Figure 1 shows the median marker levels in MoM values according to length of follow-up in affected and unaffected men. Total PSA shows the greatest discrimination; the median MoM in affected men was 14.4 in those who died of, or with, prostate cancer within five years compared to 1.0 MoM in unaffected men. The median MoM values in men who died of, or had a registration of, prostate cancer declined respectively to 3.7, 2.4, 1.7, and 1.4 for 20 + years with increasing 5-year intervals of follow-up. The next most discriminatory marker was intact PSA followed by free PSA and then hK2. There was no statistically significant discrimination in MSP MoM values for any length of follow-up and therefore this marker is not considered further. Allowing for length of follow-up, there were no statistically significant changes in MoM values for free PSA, total PSA, intact PSA or hK2 in affected men according to age, as well as, by definition, in unaffected men.

The PSA markers and hK2 MoM values showed approximately log Gaussian distributions both in affected and unaffected men with follow-up of up to 5 years and 10 years (see Figures S2 and S3). Tables S1 and S2 show the distribution parameters of these markers in affected and unaffected men (means, standard deviations and truncation limits in Table S1, correlation coefficients in Table S2). Total, free and intact PSA (log) MoM values were highly correlated in affected and unaffected men. There was less correlation between hK2 and total, free or intact PSA (log) MoM values.

Table 3 shows detection rates for specified false-positive rates of marker values expressed in mass units (ng/ml) and in MoM values. The table shows higher detection rates for specified false-positive rates when the marker values are expressed in MoM values compared with mass units (ng/ml). For example, with a 3% false-positive rate the detection rates were 90% v 88% with a follow-up of ≤5 years or 49% v 40% with a follow-up of ≤10 years. Intact PSA and hK2 show the biggest improvement in screening performance with the use of MoM values, for example at a 3% false-positive rate the use of MoM values increases the detection rate of hK2 from 35% to 45%. Table S3 shows detection rates for a greater range of specified false-positive rates. Table S4 shows, in a similar way to Table 3, false-positive rates for specified detection rates. At a 90% detection rate the false-positive rate using total PSA in MoMs was 2.8% compared to 4.5% in mass units (cut-off 3.1 ng/ml).

**Table 3.** Prostate cancer (died of, or with a history of) detection rates (DRs) for specified false-positive rates (FPRs) of individual markers expressed in mass units (ng/ml) and as age-specific multiples of the unaffected median (MoM) for the same age according to marker and length of follow-up.

	Marker in mass units (ng/ml)				Marker expressed in MoM values			
	DR (%) (95% CI) for FPR of:-				DR (%) (95% CI) for FPR of:-			
Screening marker	1%	3%	5%	10%	1%	3%	5%	10%
Follow-up ≤5 years								
Free PSA	58 (24–88)	71 (35–95)	76 (40–97)	84 (50–99)	64 (16–90)	73 (23–94)	78 (27–96)	83 (35–98)
Total PSA	82 (34–99)	88 (44–100)	90 (51–100)	93 (59–100)	86 (41–99)	90 (51–100)	92 (55–100)	95 (63–100)
Intact PSA	48 (15–80)	64 (28–91)	71 (35–96)	81 (46–99)	62 (25–93)	73 (31–96)	78 (36–98)	85 (45–100)
hK2	19 (0–58)	35 (0–73)	46 (5–83)	62 (18–92)	28 (0–65)	45 (4–81)	55 (9–88)	69 (25–96)
Follow-up <10 years								
Free PSA	13 (4–32)	24 (10–46)	32 (16–55)	44 (28–66)	23 (9–47)	34 (17–59)	40 (22–65)	51 (32–74)
Total PSA	26 (9–45)	40 (19–59)	49 (28–66)	62 (44–76)	36 (16–52)	49 (28–63)	56 (36–69)	66 (48–77)
Intact PSA	12 (3–28)	22 (8–41)	29 (13–48)	41 (22–60)	20 (6–38)	30 (13–49)	36 (17–56)	46 (26–65)
hK2	12 (4–26)	22 (11–38)	29 (17–46)	41 (30–59)	14 (5–28)	24 (13–39)	30 (18–47)	41 (27–56)

PSA: prostate specific antigen; hK2: human kallikrein-2.

**Figure 2.**
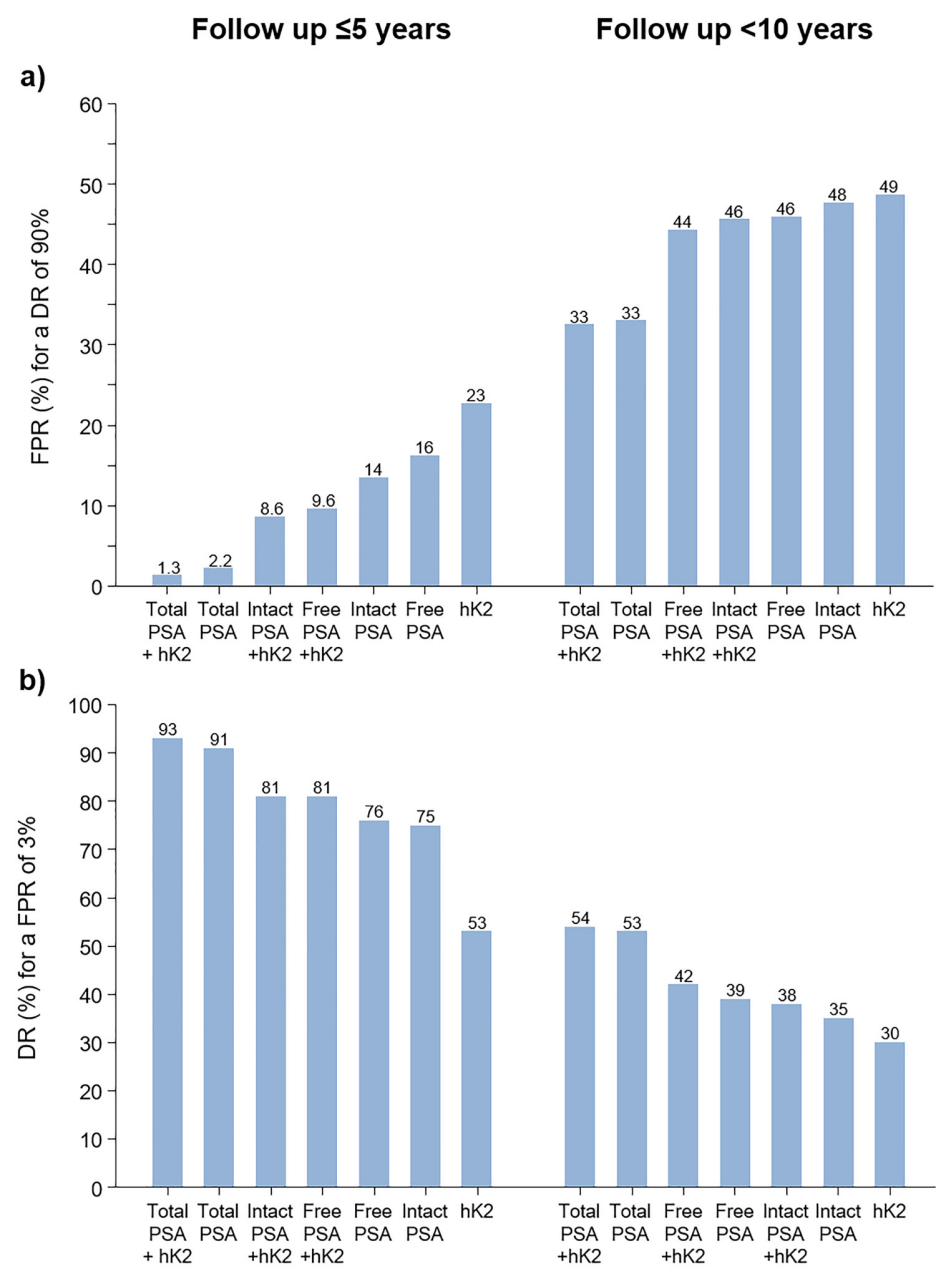
Prostate cancer (died of, or with a history of) screening performance expressed as the false-positive rate (FPR) for a 90% detection rate (DR) (a) and the DR for a 3% FPR (b) using single markers expressed in multiple of the median values together with age according to marker(s) and length of follow-up. PSA: Prostate specific antigen; hK2: Human kallikrein-2.

Figure 2A shows false-positive rates for a 90% detection rate for single markers and for combinations of markers with age according to length of follow-up of ≤5 years and <10 years. Total PSA and hK2 and age in combination yielded a 1.3% false-positive rate for a 90% detection rate compared with a false-positive rate of 2.2% and 23%, respectively, for total PSA and hK2 separately with a follow-up ≤5 years. Table S5 shows the false-positive rates for a range of detection rates (not just 90%).

Figure 2B shows, in a similar way to Figure 2A, detection rates for a 3% false-positive rate. The most discriminatory combination of markers was total PSA and hK2, with a 93% detection rate with a follow-up ≤5 years and a 54% detection rate with a follow-up <10 years. Table S5 shows detection rates for a range of false-positive rates.

Table 4 shows the detection and false-positive rates according to mass unit cut-offs (from 1 to 7 ng/ml) for total PSA and, for given detection rates, the false-positive rates based on the risk-based screening algorithm that combines total PSA and hK2 with age. Using the algorithm the false-positive rates were between about 40% and 70% lower; for example using a cut-off of 4 ng/ml the detection rate for total PSA alone was 86% and the false-positive rate was 2%, but for the same 86% detection rate the false-positive rate using the screening algorithm was 0.5%.

**Table 4.** Prostate cancer (died of, or with a history of) detection rate (DR) and false-positive rate (FPR) for total PSA mass units alone according to specified total PSA cut-off, and the FPR for the same DR using the multivariate screening algorithm based on total PSA with hK2 and age.

Total PSA alone			Screening algorithm
Cut-off (ng/ml)	DR (%)	FPR (%)	FPR (%) for the same DR
2	94.9	14.5	5.5
3	90.5	5.1	1.4
4	86.0	2.0	0.5
5	81.6	0.9	0.3
6	77.4	0.4	0.2
7	73.5	0.2	0.1

PSA: prostate specific antigen; hK2: human kallikrein-2.

Tables 3 and 4, and Figure 2 taken together show that screening performance is increased by (i) expressing marker values in age-specific MoM values instead of ng/ml and (ii) including age in the screening algorithm. For example at a 3% false-positive rate the detection rate for total PSA MoM with age is 93% (Figure 2) but without age is 90% (Table 3), and without expressing total PSA in MoM values is 88% (Table 3).

**Table 5.** Prostate cancer (died of, or with a history of) screening performance (detection rate [DR], false-positive rate [FPR] and odds of becoming affected given a positive test results [OAPR; 1:x] according to specified risk cut-off*) using single markers and markers in combination (all with age) according to markers used and length of follow-up.

	Detection rate (%) (95% CI) and false-positive rate (%) (95% CI) for risk cut-off of:-														
	1 in 10			1 in 20			1 in 30			1 in 40			1 in 50		
	DR	FPR	OAPR	DR	FPR	OAPR	DR	FPR	OAPR	DR	FPR	OAPR	DR	FPR	OAPR
≤5 years follow-up															
Free PSA	69 (25 to 92)	1.4 (1 to 3.1)	1.4	76 (38 to 93)	3 (1.7 to 6.9)	2.7	80 (42 to 94)	4.6 (2.4 to 11.6)	3.9	82 (54 to 95)	6.1 (3.1 to 19.6)	5.0	84 (61 to 96)	7.7 (3.7 to 30)	6.2
Total PSA	83 (30 to 96)	.7 (.1 to 1.8)	0.6	88 (49 to 98)	1.4 (.3 to 4.6)	1.1	90 (55 to 98)	2.1 (.4 to 7.8)	1.6	91 (60 to 99)	2.8 (.5 to 11.4)	2.1	92 (63 to 99)	3.4 (.6 to 15)	2.5
Intact PSA	66 (31 to 91)	1.5 (.9 to 2.9)	1.5	76 (44 to 94)	3.3 (1.5 to 6)	2.9	81 (53 to 95)	5.1 (1.9 to 9.8)	4.2	84 (59 to 96)	6.7 (2.5 to 14.8)	5.4	86 (67 to 97)	8.2 (3 to 20.8)	6.4
hK2	54 (15 to 86)	3.2 (1.7 to 12.4)	4.0	70 (42 to 91)	7.1 (3.3 to 15.6)	6.8	77 (54 to 93)	10.5 (4.9 to 20.8)	9.2	82 (61 to 95)	13.4 (6 to 25.4)	11.0	85 (67 to 96)	16.1 (7.1 to 29.9)	12.8
Free PSA & hK2	75 (31 to 92)	1.3 (.6 to 6.2)	1.2	81 (43 to 93)	2.8 (1.4 to 12.2)	2.3	84 (49 to 93)	4.2 (2.1 to 15.9)	3.4	86 (59 to 94)	5.4 (2.5 to 19.4)	4.2	87 (68 to 94)	6.7 (3 to 25.2)	5.2
Total PSA & hK2	87 (50 to 97)	.6 (.1 to 2.1)	0.5	90 (56 to 98)	1.2 (.2 to 4.7)	0.9	91 (64 to 98)	1.8 (.3 to 8.5)	1.3	92 (68 to 99)	2.4 (.5 to 11.8)	1.8	93 (66 to 99)	2.9 (.5 to 14)	2.1
Intact PSA & hK2	74 (38 to 93)	1.4 (.7 to 3.4)	1.3	81 (48 to 94)	2.9 (1.3 to 7.8)	2.4	85 (52 to 95)	4.4 (1.7 to 12)	3.5	87 (59 to 95)	5.7 (2.1 to 16.8)	4.4	88 (59 to 96)	6.9 (2.5 to 20.1)	5.3
<10 years follow-up															
Free PSA	50 (34 to 71)	6.1 (4.6 to 9.2)	3.4	68 (58 to 78)	16.8 (11.4 to 26.4)	6.9	79 (71 to 82)	28.4 (16.9 to 43.9)	10.0	87 (84 to 91)	41.3 (23.5 to 56.4)	13.2	92 (89 to 95)	50.5 (28.4 to 63.3)	15.2
Total PSA	63 (46 to 72)	5.7 (4 to 7.9)	2.5	76 (65 to 83)	13.1 (9.6 to 19.4)	4.8	83 (77 to 88)	20.3 (15.2 to 30.8)	6.8	87 (83 to 91)	26.5 (20.1 to 42.8)	8.4	90 (87 to 92)	32.2 (23.5 to 51.6)	9.9
Intact PSA	46 (22 to 61)	6.1 (4.3 to 8.8)	3.7	66 (53 to 74)	18.1 (12.7 to 28.7)	7.6	80 (69 to 83)	32.3 (20.3 to 48.5)	11.2	89 (85 to 92)	45.6 (29.2 to 60)	14.2	93 (90 to 95)	54.3 (35.4 to 66.6)	16.2
hK2	44 (29 to 57)	7.3 (5 to 10.9)	4.6	68 (57 to 76)	21.5 (15.2 to 29.7)	8.8	82 (76 to 86)	36.1 (26 to 47.5)	12.2	90 (87 to 93)	48.6 (35.1 to 58.6)	15.0	94 (91 to 95)	56.8 (41.8 to 65.2)	16.8
Free PSA & hK2	52 (32 to 65)	6.2 (4.6 to 9.3)	3.3	69 (54 to 76)	16.2 (11.2 to 23.1)	6.5	79 (69 to 83)	26.8 (18.5 to 40.9)	9.4	86 (78 to 90)	37.2 (26 to 54.7)	12.0	91 (88 to 95)	46.8 (34.3 to 63.3)	14.3
Total PSA & hK2	64 (47 to 71)	5.7 (4.2 to 8.2)	2.5	76 (65 to 83)	13.1 (9.7 to 19.6)	4.8	83 (75 to 88)	20 (15.1 to 30.5)	6.7	87 (80 to 91)	26.1 (19.7 to 42)	8.3	90 (84 to 93)	31.7 (23.4 to 51.2)	9.8
Intact PSA & hK2	49 (25 to 60)	6.4 (4.7 to 9.2)	3.6	68 (56 to 75)	17.5 (12.7 to 27.1)	7.1	80 (68 to 83)	29.1 (20.4 to 46.2)	10.1	87 (81 to 91)	40.4 (29.7 to 57.8)	12.9	92 (89 to 95)	49.7 (37.7 to 64.6)	15.0

*Risk relates to the specified period of follow-up. PSA = prostate specific antigen; hK2 = human kallikrein-2.

Minor differences between results in this Table and results in Figure 2 and Table S5 are due to rounding arising from fixing the DR of FPR, as in Figure 2 and Table S5, or fixing the risk cut-off as in this Table.

Table 5 shows detection rates, false-positive rates and odds of becoming affected given a positive test according to specified risk cut-offs for total PSA with age and total PSA and hK2 expressed in MoMs and combined with age; with a follow-up of ≤5 years. At a 1 in 20 risk cut-off, using total PSA and hK2 together with age yields a 90% detection rate for a 1.2% false-positive rate and an odds of becoming affected over 5 years of 1:0.9.

**Figure 3.**
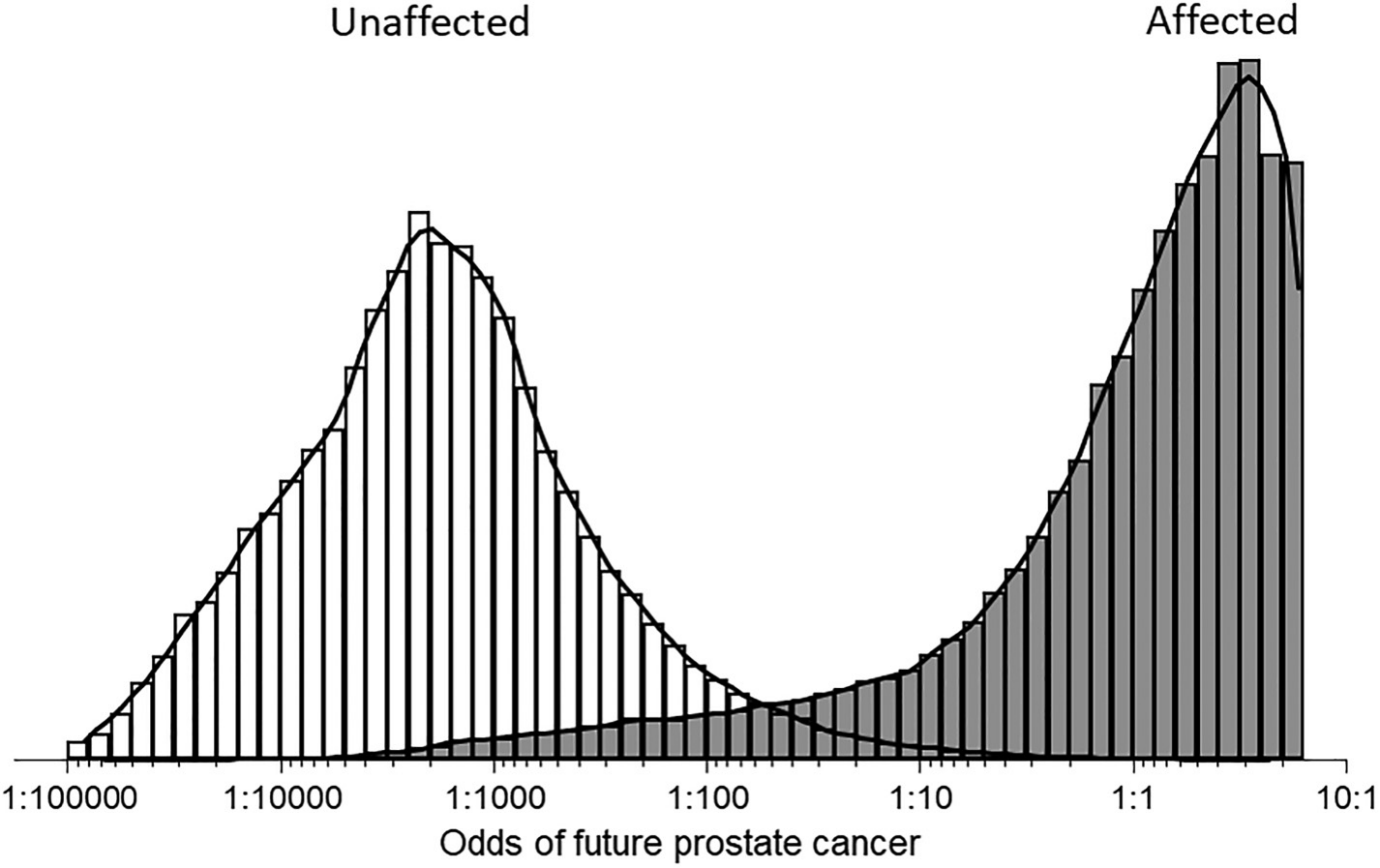
Distributions of risk estimates based on total prostate specific antigen and human kallikrein-2 multiple of the median values combined with age in simulated populations of men who did (affected) and did not (unaffected) die of, or with, a prostate cancer diagnosis up to 5 years later. (The maximum odds ratio is about 10:1 because of marker level truncation limits.).

 Figure 3 shows the distributions of risk estimates based on total PSA and hK2 combined with age with a follow-up of ≤5 years in simulated populations of men who did and did not die of or with future prostate cancer. There is wide separation between the two distributions illustrating the high screening performance of the screening algorithm.

## Discussion

### Screening performance

Our results show that a multi-marker risk-based screening algorithm incorporating a man's age, total PSA value, hK2 value, with the markers expressed in MoMs yields an improved screening performance compared to using total PSA alone. The predictive effect of PSA is similar for men whose cause of death is prostate cancer and those who have non-fatal prostate cancer. Our results show that the proposed screening algorithm used to identify men with a 5-year risk of 1 in 20 (5%) achieves a 90% detection rate for a 1.2% false-positive rate, which yields an odds of becoming affected over 5 years given a positive result of about 1:1 (50%).

In this study, men who developed prostate cancer were matched with men who did not die with, or of, prostate cancer according to age, duration of storage of serum samples and number of freeze-thaw cycles to ensure comparability. The screening algorithm incorporates the discrimination of age, with the age-specific rates taken from Cancer Research UK cancer statistics based on national data; age data from our study could not be used because cases and controls were age-matched. Although hK2 alone is a relatively weak screening marker for prostate cancer it is relatively independent of PSA so the two together yield a better test than PSA alone. Our results show that MSP however is not a useful screening marker for prostate cancer, and given the performance of total PSA, adding free PSA or intact PSA, which are highly correlated with total PSA, has a negligible effect on screening performance.

It is, perhaps, surprising that in spite of a large literature on prostate cancer screening using PSA and its sub units there is a notable absence of studies that present results that properly evaluate the performance of the screening tests, namely in terms of detection rates for given false-positive rates or *vice versa*. Often odds ratios are reported for either a one unit or one standard deviation increase in the screening marker, or by comparing groups such as the upper versus lower quintile of marker values. These fail to provide any direct measure of screening performance. While there is a numerical equivalence between an odds ratio and the detection rate for a specified false-positive rate, or false-positive rate for a specified detection rate, odds ratios need to be very large for them to translate to a useful screening test.^12,13^ The area under the receiver operating characteristic (ROC) curve (AUC) is often reported, but values are difficult to interpret, can be misleading and do not provide what is needed in practice.^14^ For example, the AUC covers the whole range of values from 0 to 100% detection when only a small portion of the ROC curve is relevant. A higher AUC does not necessarily mean a better screening test at the point at which the false-positive rate would be acceptable; one screening test can have a higher AUC than another test, but a lower detection rate for a given false-positive rate. In this study we provide direct estimates of screening performance using biochemical markers considered in past work, individually and in combination, together with a man's age, all of which are needed to guide screening policy.

The study shows the limitation of using a fixed PSA cut-off level (e.g. 4 ng/ml) that does not take account of the steep rise of PSA with age, something that can be readily solved by expressing PSA values in MoMs. Using a fixed ng/ml cut-off of 4 ng/ml, the detection and false-positive rates were 86% and 2% respectively for a follow-up of ≤5 years, a false-positive rate about 4 times higher than when using MoMs and age and including hK2 in the screening algorithm. The improvement in screening performance is worthwhile. The approach we have used in our multi-marker screening method, while innovative in cancer screening, is widely used in prenatal screening for Down syndrome, which relies on several markers and uses MoM values to take account of gestational age^11,15^ instead of a man's age as we describe here in prostate cancer screening. This screening method has the advantage that should new markers be identified in the future, they can easily be incorporated into the screening algorithm, as can existing marker parameters be updated in the light of new information.

It has been shown that PSA levels are lower in men who have a higher body mass index (BMI).^16,17^ Conceptually one could allow for the increase in a screening algorithm in the same way that we used MoM values to allow for the increase in marker levels with increasing age. In our data, all screening markers statistically significantly decreased with increasing BMI (*p* < 0.001), apart from total PSA which was of borderline statistical significance (*p* = 0.052; see Figure S4). There is negligible difference in screening performance whether or not MoM values are adjusted for BMI; for example at a 2% false-positive rate, the detection rates for the combination of total PSA and hK2 (with age) are 91.8% and 91.9% without and with BMI adjustment respectively; at an 80% detection rate, respective false-positive rates are 2.5% and 2.4%; so there is little advantage in allowing for BMI in the screening algorithm. The relevant screening marker parameters for BMI-adjusted MoM values are given in Tables S6 and S7, and Tables S8 and S9 compare screening performance with and without adjusting for BMI.

The samples used in this study had been stored for up to about 30 years. While this may have caused degradation of the markers measured, cases and controls were matched for duration of sample storage so any relative differences in marker values between affected and unaffected men is expected to be maintained. The use of MoM value allows for a person's age but also analytical differences between assays or laboratories, and the effects of sample handling and duration of storage. For example, if the marker levels were, on average, half of what they would have been had the assays been performed immediately after blood collection, the absolute difference in average levels between affected and unaffected men would be half of what they should be but the relative difference would be the same; by using MoMs the relative differences are used in the analysis, not the absolute differences.

A limitation of this study is the small number of cases of prostate cancer with 5 or less years of follow-up which leads to wide confidence intervals around estimates of screening performance. However, the median PSA MoM values are consistent with the pooled results from an earlier publication based on the results from four cohort studies (the BUPA study with data used in this study, the US CLUE study, the Finnish North Karelia study and the Finnish Social Insurance Institution study).^3^ The PSA MoM values in cases with <3 and 3–5 years of follow-up were 23 and 4 respectively compared with 14.4 in this study, indicating that our results are reasonably robust Also, beyond 5 years of follow-up there is still discrimination in PSA between men who died of or with a history of prostate cancer and men who did not, with an exponential decrease in MoM values, adding weight to the observed high PSA MoM values observed up to 5 years follow-up. Another limitation is having only baseline measurements of the biochemical markers. Serial measurements would have allowed the assessment of the rate of change of markers in detecting future prostate cancer, which may improve screening performance.

### Impact on prostate cancer mortality

Although we have shown that the screening performance of PSA was improved by using a multi-marker risk-based algorithm, the results of this study do not provide the full information that such screening is worthwhile. It is also necessary to show that medical intervention following a positive screening test reduces mortality from prostate cancer. Four randomised controlled trials have been carried out with mixed results. The earliest trial, the Quebec trial, published in 2004, reported as the main result a 62% decrease in prostate cancer mortality after 8 years of follow-up (95% CI 25% to 81% decrease).^18^ This result was, however, based on an “on-treatment” analysis while the intention-to-treat analysis showed no statistically significant effect of PSA screening, a result that cannot be regarded as negative because there was poor adherence to the randomised allocation. The remaining three trials had longer periods of follow-up; the European ERSPC trial (16 years follow-up),^19^ the American PLCO trial (15 years)^20^ and the UK CAP trial (10 years).^21^ Neither the CAP nor PLCO trial showed that PSA screening reduced prostate cancer mortality. This is, however, not surprising because in the CAP trial 60% of men randomised to screening did not receive it, and 10–15% of men randomised to not receive screening did receive it. In the PLCO trial there was even less adherence to the randomised allocation, in which about 50% of those randomised to not receive screening had either already had it before being randomised or did so during follow-up. The ERSPC trial did not experience such poor adherence to the randomised allocation. It provides the only reliable evidence of the effect of PSA screening on prostate cancer mortality. After 16 years of follow-up, based on an intention-to-treat analysis, the reduction in prostate cancer mortality was 20% (95% CI 11% to 28%).^22^ Using an on-treatment analysis the reduction in prostate cancer mortality was 25% for men who were screened once, and 48% for men who were screened more than once (screening interval 2–4 years).

### Overdiagnosis

Overdiagnosis is a recognised problem in screening for cancers and this is particularly the case in screening for prostate cancer. Overall in our study, 43% (247/569) of men died with a history of prostate cancer, without prostate cancer being the cause of death. Among men with <10 years of follow-up the proportion was 35% (16/46) and among men with ≤5 years of follow-up it was 33% (8/12). Not all such cases will represent overdiagnosis; some will have had cancer clinically identified and successfully treated with the individuals concerned dying of causes other than prostate cancer. Among men who died with prostate cancer it is not possible to distinguish overdiagnosis cases from cases that benefitted from the early diagnosis; however, cases were likely to have been detected clinically rather than through PSA screening because systematic PSA screening has not been instituted in the UK. Even though the men may not have died of prostate cancer they would have had sufficient symptoms to seek medical attention. Our data set an upper bound of about 35% for the proportion of screen-positive overdiagnoses. The true proportion could be much lower. While screening will reduce prostate cancer mortality there is a need to consider the adverse effects of treatment in both true and false positives.^23,24^

### Screening practice

**Figure 4.**
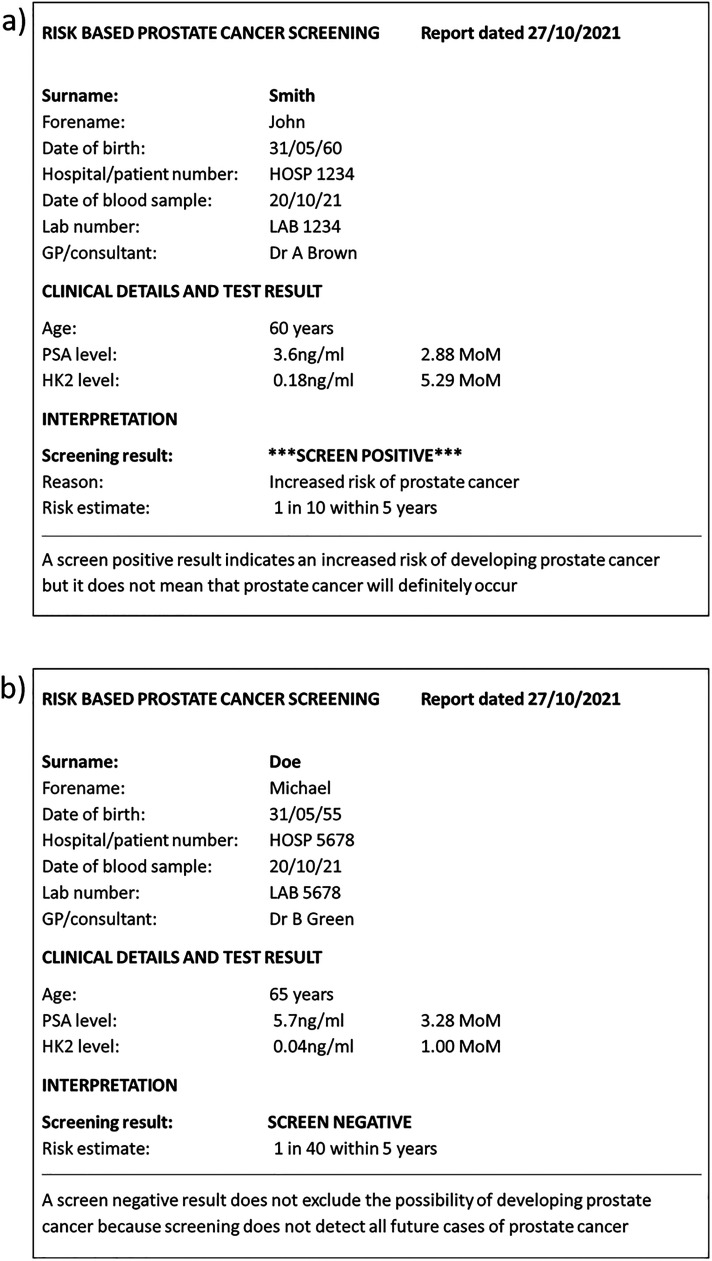
A hypothetical positive (a) and negative (b) screening report. PSA: Prostate specific antigen; hK2: Human kallikrein-2; MoM: Multiple of the median.

The ERSPC used a single PSA cut-off (3.0 ng/ml) to define men as being screen positive. With screening based on the multi-marker risk-based algorithm described in this paper, efficacy would be improved and hence the expected reduction in prostate cancer mortality would be greater than that achieved in the randomised trials, with many fewer false-positives and therefore less adverse effects from treatment. Our data indicate that a reasonable age to start screening would be 55, given that 99% (565/569) of affected men died of or with a history of prostate cancer aged 55 or over, and our data suggest a screening interval of up to 5 years, i.e. a 3-year interval would be reasonable. Figure 4 shows two examples of screening reports, one screen positive and one screen negative, which could be used in practice. Screening using the algorithm described in this paper would, using a risk cut-off of 1 in 20 (Table 5), identify about 90% of future deaths from, or with a history of, prostate cancer within a 5-year timeframe that would otherwise occur with a 1.2% false-positive rate. The effect on prostate cancer mortality would be expected to be greater than the 20% observed in the ERSPC trial because the multi-marker risk-based screening algorithm is likely to detect more cases than PSA alone.

Our data permitted an analysis of the effect of a “once-only” PSA test in which the risk may be so low that further periodic testing was unnecessary. We examined this strategy by identifying men with risks of 1 in 5000 or less, observing the number of missed cases at the time and the number of periodic screening examinations avoided in unaffected men. Over the intervals 0–5, 6–9, 11–14, 15–19 and 20 + years, the proportion of men in whom prostate cancer would be missed was 0%, 3%, 13%, 18% and 36% respectively, and the corresponding proportion of screening examinations avoided in unaffected men was respectively 30%, 27%, 30%, 34% and 47%. This analysis shows that even a very low risk of prostate cancer at an initial screening examination does not exclude the risk of having prostate cancer later. Screening performance could be improved by taking account of a previous result when interpreting a subsequent result, as has been adopted in antenatal screening for Down's syndrome.^25^

### Long-term biological implication

Of biological interest is the observation that PSA levels in affected men were statistically significantly elevated prior to their diagnosis of or death from prostate cancer, by as much as 30 years when the mean PSA was about 40% higher, indicating that the inception of prostate cancer arises many years before clinical presentation. However, beyond five years the magnitude of the predictive effect is too small to be of value in screening, and certainly too small after 10 years.

## Conclusion

Our results show that if screening is to be carried out it should be done using a multi-marker risk-based screening algorithm instead of a fixed PSA cut-off level. At minimal extra cost this would achieve a high detection rate and, importantly, compared with conventional PSA-only based screening would reduce the false-positive rate by about three-quarters. This, in turn, would reduce the number of unnecessary prostate biopsies and operations, and reduce the overdiagnosis and overtreatment associated with prostate cancer screening.

## Supplemental Material

sj-pdf-1-msc-10.1177_09691413221076415 - Supplemental material for Multi-marker risk-based screening for prostate cancer Click here for additional data file. Supplemental material, sj-pdf-1-msc-10.1177_09691413221076415 for Multi-marker risk-based screening for prostate cancer by Nicholas J Wald, Jonathan P Bestwick and Joan K Morris in Journal of Medical Screening

## References

[1] Office for National Statistics. Deaths from prostate cancer, England and Wales, 2001 to 2017. Available from https://www.ons.gov.uk/peoplepopulationandcommunity/birthsdeathsandmarriages/deaths/adhocs/009669deathsfromprostatecancerenglandandwales2001to2017. [Accessed 07/05/20]

[2] U.S. Cancer Statistics Working Group. U.S. Cancer statistics data visualizations tool, based on 2019 submission data (1999–2017): U.S. Department of Health and Human Services, Centers for Disease Control and Prevention and National Cancer Institute; www.cdc.gov/cancer/dataviz, released in June 2020.

[3] Parkes C Wald NJ Murphy P , et al. Prospective observational study to assess value of prostate specific antigen as screening test for prostate cancer. Br Med J 1995; 311: 1340–1343. 7496284 10.1136/bmj.311.7016.1340 PMC2551245

[4] Moyer VA . Screening for prostate cancer: U.S. Preventive services task force recommendation statement. Ann Intern Med. 2012; 157: 120–134. 22801674 10.7326/0003-4819-157-2-201207170-00459

[5] Yousef GM Diamandis EP . The new human tissue kallikrein gene family: structure, function, and association to disease. Endoc Rev 2001; 22: 184–204. 10.1210/edrv.22.2.0424 11294823

[6] Shariat SF Semjonow A Lilja H , et al. Tumor markers in prostate cancer I: blood-based markers. Acta Oncol (Madr) 2011; 50: 61–75. 10.3109/0284186X.2010.542174 PMC3571678 21604943

[7] Haiman CA Stram DO Vickers AJ , et al. Levels of beta-microseminoprotein in blood and risk of prostate cancer in multiple populations. J Natl Cancer Inst 2013; 105: 237–243. 23213189 10.1093/jnci/djs486 PMC3565627

[8] Parekh DJ Punnen S Sjoberg D , et al. A multi-institutional prospective trial in the USA confirms that the 4Kscore accurately identifies men with high-grade prostate cancer. Eur Urol 2015; 68: 464–470. 25454615 10.1016/j.eururo.2014.10.021

[9] Sjoberg DD Vickers AJ Assel M , et al. Twenty-year risk of prostate cancer death by midlife prostate- specific antigen and a panel of four kallikrein markers in a large population-based cohort of healthy men. Eur Urol 2018; 73: 941–948. 29519548 10.1016/j.eururo.2018.02.016 PMC5960423

[10] Wald NJ Watt HC George L , et al. Adding free to total prostate-specific antigen levels in trial of prostate cancer screening. Br J Cancer 2000; 82: 731–736. 10682690 10.1054/bjoc.1999.0988 PMC2363306

[11] Wald NJ Cuckle HS Densem JW , et al. Maternal serum screening for down’s syndrome in early pregnancy. Br Med J 1988; 297: 883–887. 2460174 10.1136/bmj.297.6653.883 PMC1834444

[12] Wald NJ Hackshaw AK Frost CD . When can a risk factor be used as a worthwhile screening test? Br Med J 1999; 319: 1562. 10591726 10.1136/bmj.319.7224.1562 PMC1117271

[13] Wald NJ Morris JK . Assessing risk factors as potential screening tests: a simple assessment tool. Arch Int Med 2011; 171: 286–291. 20975013 10.1001/archinternmed.2010.378

[14] Wald NJ Bestwick JP . Is the area under an ROC curve a valid measure of the performance of a screening or diagnostic test? J Med Screen 2014; 21: 51–56. 24407586 10.1177/0969141313517497

[15] Wald NJ Bestwick JP Huttly WJ . Improvements in antenatal screening for down’s syndrome. J Med Screen 2013; 20: 7–14. 23512549 10.1177/0969141313476496 PMC5438079

[16] Rundle A Neugut AI . Obesity and screening PSA levels among men undergoing an annual physical exam. Prostate. 2008; 68: 373–380. 18189231 10.1002/pros.20704

[17] Culp S Porter M . The effect of obesity and lower serum prostate-specific antigen levels on prostate-cancer screening results in American men. BJU Int. 2009; 104: 1457–1461. 19522868 10.1111/j.1464-410X.2009.08646.x

[18] Labrie F Candas B Cusan L , et al. Screening decreases prostate cancer mortality: 11-year follow-up of the 1988 Quebec prospective randomized controlled trial. Prostate 2004; 59: 311–318. 15042607 10.1002/pros.20017

[19] Hakama M Moss SM Stenman UH , et al. Design-corrected variation by centre in mortality reduction in the ERSPC randomised prostate cancer screening trial. J Med Screen 2017; 24: 98–103. 27510947 10.1177/0969141316652174 PMC5299065

[20] Pinsky PF Prorok PC Yu K , et al. Extended mortality results for prostate cancer screening in the PLCO trial with median follow-up of 15 years. Cancer 2017; 123: 592–599. 27911486 10.1002/cncr.30474 PMC5725951

[21] Martin RM Donovan JL Turner EL , et al. For the CAP trial group. Effect of a low-intensity PSA-based screening intervention on prostate cancer mortality. JAMA 2018; 319: 883–895. 29509864 10.1001/jama.2018.0154 PMC5885905

[22] Hugosson J Roobol MJ Mansson M , et al. A 16-yr follow-up of the European randomized study of screening for prostate cancer. Eur Urol 2019; 76: 43–51. 30824296 10.1016/j.eururo.2019.02.009 PMC7513694

[23] Shoag JE Nyame YA Gulati R , et al. Reconsidering the trade-offs of prostate cancer screening. N Engl J Med 2020; 382: 2465–2468. 32558473 10.1056/NEJMsb2000250 PMC7491201

[24] Welch HG Albertsen PC . Reconsidering prostate cancer mortality – the future of PSA screening. N Engl J Med 2020; 382: 1557–1563. 32294352 10.1056/NEJMms1914228

[25] Wald NJ Huttly WJ Rudnicka AR . Prenatal screening for down syndrome: the problem of recurrent false-positives. Prenat Diagn. 2004; 24: 389–392. 15164416 10.1002/pd.890

