# Supplementary material for: Multi-marker risk-based screening for prostate cancer
Source: J Med Screen. 2022 Mar 7;29(2):123–33. doi: 10.1177/09691413221076415 (PMC9087319; doi:10.1177/09691413221076415)
Supplement: sj-pdf-1-msc-10.1177_09691413221076415 - Supplemental material for Multi-marker risk-based screening for prostate cancer [file sj-pdf-1-msc-10.1177_09691413221076415.pdf]

Multi-marker risk-based screening for prostate cancer: Supplementary appendix

Biochemical analyses

Total and free PSA levels were measured at Lund University, Malmö Sweden using the AutoDelfia 1235 automatic immunoassay system using the dual-label DELFIA Prostatus total/free PSA-Assay (Perkin-Elmer, Turku, Finland) calibrated against the World Health Organization (WHO) 96/670 (PSA-WHO) and WHO 68/668 (free PSA-WHO) standards. Intact PSA and hK2 were measured with F(ab')2 fragments of the monoclonal capture antibodies to reduce the frequency of nonspecific assay interference, as described in detail previously[1]. The production and purification of the polyclonal rabbit anti-MSMB antibody, protocols for biotinylation and Europium labeling of the anti-MSMB antibody, and performance of the MSMB immunoassay were carried out as previously reported[2].

Statistical methods

Marker levels were converted into multiple of the median (MoM) values by first performing median regressions of marker levels against age among unaffected men and then dividing observed marker levels by that expected from the median regression. Length of follow-up was categorised into five groups: 0-5 years, 6-9 years, 10-14 years, 15-19 years and 20+ years between blood sampling and death or cancer registration. Wilcoxon rank sum tests were used to compare marker MoM values between affected and unaffected men, and within follow-up categories. For each marker the median was calculated and distributions were assessed using probability plots (plots of observed MoM values against centiles, with centiles on a z-score scale), separately in affected and unaffected men to assess if the marker MoM values followed a reasonable fit to Gaussian distributions, after log transformation. Robust standard deviations of the $\log_{10}$ marker values in affected and unaffected men were estimated as the slope from a regression of the points on the probability plots, between the 10th and 90th centiles. Correlation coefficients of the $\log_{10}$ marker values were calculated after excluding observations more the 3.5 standard deviations from the log of the median. Truncation limits were specified by inspection of the probability plots at either the point of deviation from linearity (i.e. where the data deviate from the Gaussian distribution), where data run out, or at the point of risk reversal (which occurs when the standard deviation in affected and unaffected men differ to the extent that for increasing (or decreasing) values of the marker the likelihood ratio (height of the curve in in affected men divided by the height of the curve in unaffected men) changes direction.[3] Marker MoM values lower (or higher) than the specified lower (or

higher) truncation limits were truncated at the lower (or higher) limit. The medians, standard deviations, correlation coefficients (log_{10}) and truncation limits (herein referred to as parameters) define the multivariate Gaussian distributions of markers in affected and unaffected men. The prior risk of prostate cancer was based on the incidence per 100,000 men per year of prostate cancer according to age groups in the UK 2015-2017, taken from Cancer Research UK cancer statistics[4]:

Age group	Incident Cases	Incidence (per 100,000 men per year)	Age group	Incident Cases	Incidence (per 100,000 men per year)
0 to 4	1	0.05	50 to 54	1585	70.38
5 to 9	0	0	55 to 59	3695	189.43
10 to 14	0	0	60 to 64	5823	339.61
15 to 19	1	0.05	65 to 69	10143	577.97
20 to 24	0	0	70 to 74	9845	759.43
25 to 29	0	0	75 to 79	8140	820.69
30 to 34	2	0.09	80 to 84	4804	707.62
35 to 39	8	0.39	85 to 89	2725	743.11
40 to 44	80	3.76	90+	1192	728.97
45 to 49	442	19.37			

Risk according to age (expressed as an odds) was estimated using negative exponential regression and is shown in the following figure

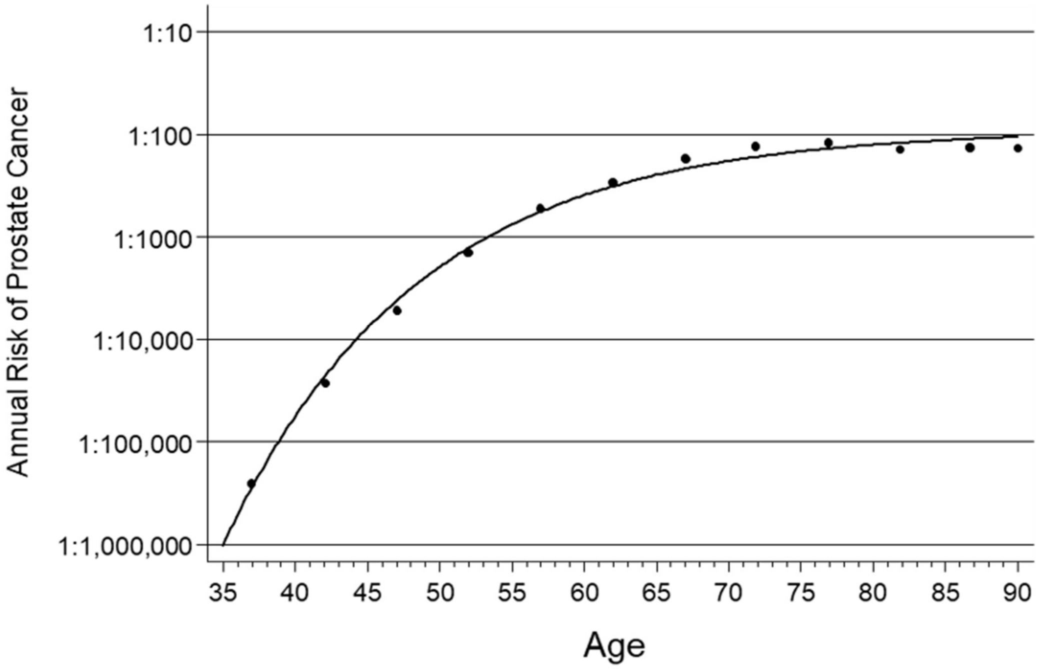

The annual odds of prostate cancer according to age is given by the following equation

$$annual\ odds\ of\ prostate\ cancer = 10^{1.960998+0.6376318\times \exp(-0.073928\times(age-60))}$$

Five and 10 year risks of prostate cancer were calculated by annual summation.

Estimates of the screening performance for each marker, and marker combinations was estimated by simulating 100,000 affected and 100,000 unaffected men, each with marker values from the overlapping multivariate Gaussian distributions of the marker MoM values and an age based on the annual odds of prostate cancer and the mid-2016 age distribution of males in the UK[5], limited to males aged 45 or more since the incidence of prostate cancer is low at younger ages:

Age	N	Age	N	Age	N	Age	N	Age	N
45	444995	55	411759	65	340422	75	215612	85	98571
46	455570	56	402345	66	348975	76	210995	86	84424
47	456082	57	393423	67	366002	77	202447	87	71190
48	464148	58	378415	68	395680	78	188015	88	61372
49	461581	59	364674	69	303869	79	174775	89	51143
50	465548	60	350167	70	292273	80	162277	90	163519
51	461048	61	349645	71	290461	81	145722		
52	452399	62	345319	72	267272	82	133787		
53	443862	63	333812	73	236273	83	124667		
54	429088	64	335678	74	210093	84	112442		

For each simulated man, and for the various combinations of markers the likelihood ratio was calculated and multiplied by the age-specific five or ten-year risk to obtain the marker based risk of developing prostate cancer over a given period of time. A screen-positive result was defined as the risk of developing prostate cancer within a specified time interval greater than or equal to a specified risk cut-off. Estimates of the detection rate and false-positive rate at or greater than specified risk cut-offs for specified durations of follow-up were computed. The odds of being affected given a positive result was also calculated and was based on the annual risk. We also investigated whether MoM values were influenced by body mass index (BMI) by performing median regressions of (log) MoM against BMI among unaffected men and then dividing the observed MoM values by those expected from the median regressions. The above steps were then repeated to determine whether adjusting for BMI improved screening performance.

References

1 Väisänen V, Peltola MT, Lilja H, et al. Intact free prostate-specific antigen and free and total human glandular kallikrein 2. Elimination of assay interference by enzymatic digestion of antibodies to F(ab’)2 fragments. *Anal Chem* 2006;**78**:7809-15

2 Haiman CA, Stram DO, Vickers AJ, et al. Levels of beta-microseminoprotein in blood and risk of prostate cancer in multiple populations. *J Natl Cancer Inst* 2013;**105**:237-43

3 Morris JK, Wald NJ. Graphical presentation of distributions of risk in screening. *J Med Screen* 2005;**12**:155-60

4 Cancer Research UK. Prostate Cancer Incidence Statistics. Available from: https://www.cancerresearchuk.org/health-professional/cancer-statistics/statistics-by-cancer-type/prostate-cancer/incidence#heading-One. [Accessed 07/05/20]

5 Office for National Statistics. Estimates of the population for the UK, England and Wales, Scotland and Northern Ireland mid-2016. Available from: https://www.ons.gov.uk/peoplepopulationandcommunity/populationandmigration/populationestimates/datasets/populationestimatesforukenglandandwalesscotlandandnorthernireland [Accessed 07/05/20]

Table S1: Means, standard deviations and truncation limits for marker multiple of the median values in affected (died of, or with a history of, prostate cancer) and unaffected men according to length of follow-up

	Affected		Unaffected		Truncation limits	
Marker	Mean ($\log_{10}$)	SD ($\log_{10}$)	Mean ($\log_{10}$)	SD ($\log_{10}$)	Lower	Upper
≤5 years follow-up						
Free PSA	0.7772	0.4839	-0.0541	0.2831	0.4	5
Total PSA	1.1587	0.5346	-0.0401	0.2684	0.4	5
Intact PSA	0.8551	0.4775	-0.0475	0.3256	0.4	6
hK2	0.5045	0.2965	-0.0330	0.3056	0.5	5
<10 years follow-up						
Free PSA	0.3693	0.4202	-0.0208	0.2991	0.4	4
Total PSA	0.6197	0.4603	-0.0078	0.3385	0.4	6
Intact PSA	0.3808	0.4462	0.0039	0.3255	0.4	5
hK2	0.2738	0.3548	-0.0220	0.2909	0.5	5

Table S2: Correlation coefficients of log_{10} marker multiple of the median values in affected (died of, or with a history of, prostate cancer) and unaffected men according to length of follow-up

	Affected				Unaffected			
Marker	Free PSA	Total PSA	Intact PSA	hK2	Free PSA	Total PSA	Intact PSA	hK2
≤5 years follow-up								
Free PSA	1				1			
Total PSA	0.7164	1			0.8128	1		
Intact PSA	0.7453	0.7354	1		0.7525	0.7721	1	
hK2	0.4852	0.4983	0.5348	1	0.1712	0.0428	0.1782	1
<10 years follow-up								
Free PSA	1				1			
Total PSA	0.8036	1			0.8943	1		
Intact PSA	0.7813	0.7710	1		0.9575	0.8460	1	
hK2	0.6024	0.6621	0.6283	1	0.4511	0.4220	0.5169	1

Table S3: Prostate cancer (died of, or with a history of) detection rates (DRs) for specified false-positive rates (FPRs) of individual markers expressed in mass units (ng/ml) and as age-specific multiples of the unaffected median (MoM) according to length of follow-up.

	Markers in mass units (ng/ml)						Markers expressed in MoM values					
	DR (%) for FPR of:-						DR (%) for FPR of:-					
Marker	1%	2%	3%	4%	5%	10%	1%	2%	3%	4%	5%	10%
≤5 years follow-up												
Free PSA	58 (24-88)	66 (30-93)	71 (35-95)	74 (38-97)	76 (40-97)	84 (50-99)	64 (16-90)	70 (20-93)	73 (23-94)	76 (25-95)	78 (27-96)	83 (35-98)
Total PSA	82 (34-99)	86 (39-100)	88 (44-100)	89 (48-100)	90 (51-100)	93 (59-100)	86 (41-99)	89 (47-100)	90 (51-100)	91 (53-100)	92 (55-100)	95 (63-100)
Intact PSA	48 (15-80)	58 (23-88)	64 (28-91)	68 (32-94)	71 (35-96)	81 (46-99)	62 (25-93)	69 (30-95)	73 (31-96)	76 (34-97)	78 (36-98)	85 (45-100)
hK2	19 (0-58)	28 (0-67)	35 (0-73)	41 (2-79)	46 (5-83)	62 (18-92)	28 (0-65)	38 (2-75)	45 (4-81)	50 (7-86)	55 (9-88)	69 (25-96)
≤10 years follow-up												
Free PSA	13 (4-32)	20 (7-41)	24 (10-46)	28 (13-51)	32 (16-55)	44 (28-66)	23 (9-47)	30 (14-54)	34 (17-59)	38 (20-62)	40 (22-65)	51 (32-74)
Total PSA	26 (9-45)	35 (14-53)	40 (19-59)	45 (24-63)	49 (28-66)	62 (44-76)	36 (16-52)	44 (23-59)	49 (28-63)	53 (32-67)	56 (36-69)	66 (48-77)
Intact PSA	12 (3-28)	17 (5-35)	22 (8-41)	26 (10-45)	29 (13-48)	41 (22-60)	20 (6-38)	26 (9-44)	30 (13-49)	33 (15-53)	36 (17-56)	46 (26-65)
hK2	12 (4-26)	18 (8-33)	22 (11-38)	26 (15-42)	29 (17-46)	41 (30-59)	14 (5-28)	20 (9-34)	24 (13-39)	27 (15-43)	30 (18-47)	41 (27-56)

Table S4: Prostate cancer (died of, or with a history of) false-positive rates (FPRs) for specified detection rates (DRs) of individual markers expressed in mass units (ng/ml) and as age-specific multiples of the unaffected median (MoM) according to length of follow-up.

	Markers in mass units (ng/ml)					Markers expressed in MoM values				
	FPR (%) for DR of:-					FPR (%) for DR of:-				
Marker	50%	60%	70%	80%	90%	50%	60%	70%	80%	90%
≤5 years follow-up										
Free PSA	0.4 (0.0-10.2)	1.2 (0.0-21.6)	2.9 (0.1-40.2)	7.1 (0.4-62.8)	19.3 (1.4-81.7)	0.2 (0.0-25.5)	0.6 (0.0-42.2)	2.1 (0.1-67.4)	6.7 (0.3-84.5)	22.8 (1.2-95.1)
Total PSA	0.0 (0.0-3.4)	0.0 (0.0-8.8)	0.1 (0.0-20.2)	0.7 (0.0-42.7)	4.5 (0.0-73.8)	0.0 (0.0-2.3)	0.0 (0.0-6.6)	0.0 (0.0-17.9)	0.3 (0.0-43.6)	2.8 (0.0-77.8)
Intact PSA	1.2 (0.1-12.3)	2.4 (0.2-21.6)	4.6 (0.5-37.4)	9.2 (1.2-56.6)	20.2 (2.5-77.4)	0.3 (0.0-8.7)	0.8 (0.0-21.8)	2.3 (0.1-39.0)	6.2 (0.3-68.0)	18.6 (1.1-87.8)
hK2	6.1 (0.4-23.4)	9.3 (1.1-29.4)	13.9 (1.9-38.9)	21.0 (3.2-54.2)	33.9 (5.8-72.7)	3.9 (0.2-20.6)	6.5 (0.5-26.9)	10.6 (1.0-35.5)	17.3 (2.0-52.0)	30.3 (4.1-72.4)
≤10 years follow-up										
Free PSA	12.9 (3.8-24.7)	19.5 (7.0-33.7)	28.5 (12.7-45.2)	41.1 (21.6-59.9)	59.8 (37.4-78.7)	9.6 (1.4-26.5)	17.1 (3.5-38.7)	28.5 (7.9-53.6)	45.2 (16.2-70.4)	69.0 (35.6-88.7)
Total PSA	5.3 (1.6-12.8)	9.1 (3.5-19.4)	15.0 (6.9-28.6)	24.7 (12.5-42.4)	42.2 (24.9-63.5)	3.2 (0.9-10.6)	6.6 (2.4-18.2)	12.7 (5.8-29.9)	23.9 (12.1-47.2)	45.6 (26.5-74.4)
Intact PSA	14.7 (6.5-28.5)	21.7 (11.1-38.2)	31.1 (18.0-50.3)	43.9 (28.5-66.1)	62.4 (43.7-82.8)	12.3 (3.3-32.0)	20.9 (7.1-44.1)	33.0 (14.4-59.2)	49.8 (26.6-76.4)	72.5 (48.2-91.9)
hK2	15.0 (6.3-23.2)	22.4 (11.1-32.5)	32.2 (18.0-45.1)	45.5 (27.9-61.5)	64.5 (44.3-80.6)	15.5 (7.1-26.7)	24.0 (13.0-38.1)	35.3 (22.0-52.5)	50.4 (34.9-69.3)	70.8 (53.4-87.0)

Table S5: Prostate cancer (died of, or with a history of) screening performance (false-positive rate [FPR] according to specified detection rate [DR] and DR for specified FPR) using single markers and markers in combination (all with age) according to markers used and length of follow-up.

	False-positive rate (%) for detection rate of:-					Detection rate (%) for false-positive rate of:-					
	50%	60%	70%	80%	90%	1%	2%	3%	4%	5%	10%
≤5 years follow-up											
Free PSA	0.3	0.6	1.6	4.7	16.2	65	72	76	79	81	86
Total PSA	0.1	0.2	0.3	0.5	2.2	85	90	91	93	93	96
Intact PSA	0.7	1.1	2.1	4.7	13.5	58	70	75	78	81	87
hK2	2.6	4.3	7.2	12.2	22.7	34	45	53	59	63	76
Free PSA & hK2	0.1	0.3	0.8	2.5	9.6	72	78	81	84	85	90
Total PSA & hK2	0.0	0.0	0.1	0.2	1.3	89	92	93	94	95	96
Intact PSA & hK2	0.2	0.4	1.0	2.6	8.6	70	78	81	84	86	91
<10 years follow-up											
Free PSA	6.2	11.1	19	30.1	45.9	26	34	39	43	47	58
Total PSA	2.5	4.6	8.8	16.7	33	35	46	53	58	61	72
Intact PSA	7.9	13.5	21.7	32.6	47.7	21	29	35	39	42	54
hK2	9.7	15.3	23.1	33.5	48.6	18	25	30	34	38	51
Free PSA & hK2	5.3	9.8	16.9	27.6	44.3	28	37	42	46	49	60
Total PSA & hK2	2.3	4.5	8.6	16.6	32.5	38	48	54	58	62	72
Intact PSA & hK2	6.7	11.7	19	29.4	45.6	24	32	38	42	45	57

Table S6: Means, standard deviations and truncation limits for marker multiple of the median values in affected (died of, or with a history of, prostate cancer) and unaffected men according to length of follow-up. Multiple of the median values are adjusted for body mass index.

	Affected		Unaffected		Truncation limits	
Marker	Mean (log_{10})	SD (log_{10})	Mean (log_{10})	SD (log_{10})	Lower	Upper
≤5 years follow-up						
Free PSA	0.7918	0.4727	-0.0671	0.2924	0.4	5
Total PSA	1.1676	0.5232	-0.0411	0.2705	0.4	5
Intact PSA	0.8700	0.4621	-0.0474	0.3269	0.4	6
hK2	0.4965	0.2951	-0.0256	0.2931	0.5	5
<10 years follow-up						
Free PSA	0.3764	0.4158	-0.0166	0.2997	0.4	4
Total PSA	0.6257	0.4543	-0.0025	0.3379	0.4	6
Intact PSA	0.3896	0.4389	-0.0014	0.3243	0.4	5
hK2	0.2823	0.3510	-0.0288	0.2893	0.5	5

Table S7: Correlation coefficients of log_{10} marker multiple of the median values in affected (died of, or with a history of, prostate cancer) and unaffected men according to length of follow-up. Multiple of the median values are adjusted for body mass index.

	Affected				Unaffected			
Marker	Free PSA	Total PSA	Intact PSA	hK2	Free PSA	Total PSA	Intact PSA	hK2
≤5 years follow-up								
Free PSA	1				1			
Total PSA	0.7364	1			0.7719	1		
Intact PSA	0.7731	0.7615	1		0.7262	0.7543	1	
hK2	0.4947	0.5013	0.5545	1	0.2141	0.1086	0.2357	1
<10 years follow-up								
Free PSA	1				1			
Total PSA	0.7978	1			0.8814	1		
Intact PSA	0.7679	0.7668	1		0.9423	0.8373	1	
hK2	0.5983	0.6609	0.6304	1	0.4369	0.4132	0.5042	1

Table S8: Prostate cancer (died of, or with a history of) screening performance (detection rate according to specified false-positive rate) using single markers and markers in combination (all with age) according to markers used, length of follow-up, and whether multiple of the median values are unadjusted or adjusted for body mass index (BMI).

	Unadjusted: Detection rate (%) for false-positive rate of:-						BMI adjusted: Detection rate (%) for false-positive rate of:-					
	1%	2%	3%	4%	5%	10%	1%	2%	3%	4%	5%	10%
<5 years follow-up												
Free PSA	65.3	72.1	75.9	78.7	80.7	86.2	65.7	73.3	77.1	79.9	81.8	87.5
Total PSA	85.5	89.6	91.4	92.6	93.3	95.5	86.2	90.2	92.0	93.1	93.9	96.0
Intact PSA	57.8	69.6	75.0	78.3	80.9	87.4	59.1	71.1	76.5	79.7	82.3	88.7
hK2	33.9	45.4	53.2	58.8	63.0	76.4	35.3	46.4	53.9	59.2	63.3	76.3
Free PSA + hK2	72.2	78.1	81.3	83.6	85.4	90.2	72.3	78.5	81.7	84.1	85.9	90.8
Total PSA + hK2	89.0	91.8	93.1	94.1	94.6	96.5	88.9	91.9	93.3	94.2	94.8	96.7
Intact PSA + hK2	70.3	77.7	81.3	83.9	85.8	91.0	70.1	77.7	81.5	84.2	86.1	91.4
5-10 years follow-up												
Free PSA	25.7	33.8	39.2	43.4	46.7	58.1	25.6	33.8	39.2	43.5	46.9	58.4
Total PSA	34.9	46.5	53.2	57.8	61.4	72.1	33.8	46.8	53.4	58.0	61.7	72.4
Intact PSA	21.0	29.0	34.8	39.0	42.4	54.3	21.6	30.2	36.0	40.2	43.7	55.6
hK2	17.9	24.7	29.8	34.2	37.9	50.7	19.0	26.0	31.4	35.8	39.6	52.5
Free PSA + hK2	28.5	36.8	41.7	45.6	48.9	60.3	29.1	37.5	42.5	46.5	49.8	61.2
Total PSA + hK2	38.0	48.2	54.0	58.5	61.7	72.3	38.8	48.7	54.7	58.9	62.2	72.8
Intact PSA + hK2	23.9	32.3	37.7	41.7	45.2	57.2	25.4	33.9	39.3	43.4	46.9	58.9

Table S9: Prostate cancer (died of, or with a history of) screening performance (false-positive rate according to specified detection rate) using single markers and markers in combination (all with age) according to markers used and length of follow-up, and whether multiple of the median values are unadjusted or adjusted for body mass index (BMI).

	Unadjusted: False-positive rate (%) for detection rate of:-					BMI adjusted: False-positive rate (%) for detection rate of:-				
	50%	60%	70%	80%	90%	50%	60%	70%	80%	90%
<5 years follow-up										
Free PSA	0.3	0.6	1.6	4.7	16.2	0.3	0.6	1.5	4.0	13.8
Total PSA	0.1	0.2	0.3	0.5	2.2	0.1	0.2	0.3	0.5	1.9
Intact PSA	0.7	1.1	2.1	4.7	13.5	0.7	1.1	1.9	4.1	11.6
hK2	2.6	4.3	7.2	12.2	22.7	2.5	4.2	7.1	12.3	23.2
Free PSA + hK2	0.1	0.3	0.8	2.5	9.6	0.1	0.3	0.8	2.4	9.0
Total PSA + hK2	0.0	0.0	0.1	0.2	1.3	0.0	0.1	0.1	0.3	1.3
Intact PSA + hK2	0.2	0.4	1.0	2.6	8.6	0.2	0.5	1.0	2.5	8.2
5-10 years follow-up										
Free PSA	6.2	11.1	19.0	30.1	45.9	6.1	11.0	18.6	29.6	45.6
Total PSA	2.5	4.6	8.8	16.7	33.0	2.5	4.5	8.6	16.4	32.3
Intact PSA	7.9	13.5	21.7	32.6	47.7	7.3	12.6	20.5	31.4	47.0
hK2	9.7	15.3	23.1	33.5	48.6	8.9	14.2	21.8	32.1	47.7
Free PSA + hK2	5.3	9.8	16.9	27.6	44.3	5.1	9.3	16.2	26.7	43.3
Total PSA + hK2	2.3	4.5	8.6	16.6	32.5	2.2	4.3	8.4	16.1	31.8
Intact PSA + hK2	6.7	11.7	19.0	29.4	45.6	6.0	10.7	17.7	28.1	44.4

Figure S1: Median marker levels (and 95% confidence intervals) in unaffected men according to age in 5 year categories, together with regression lines

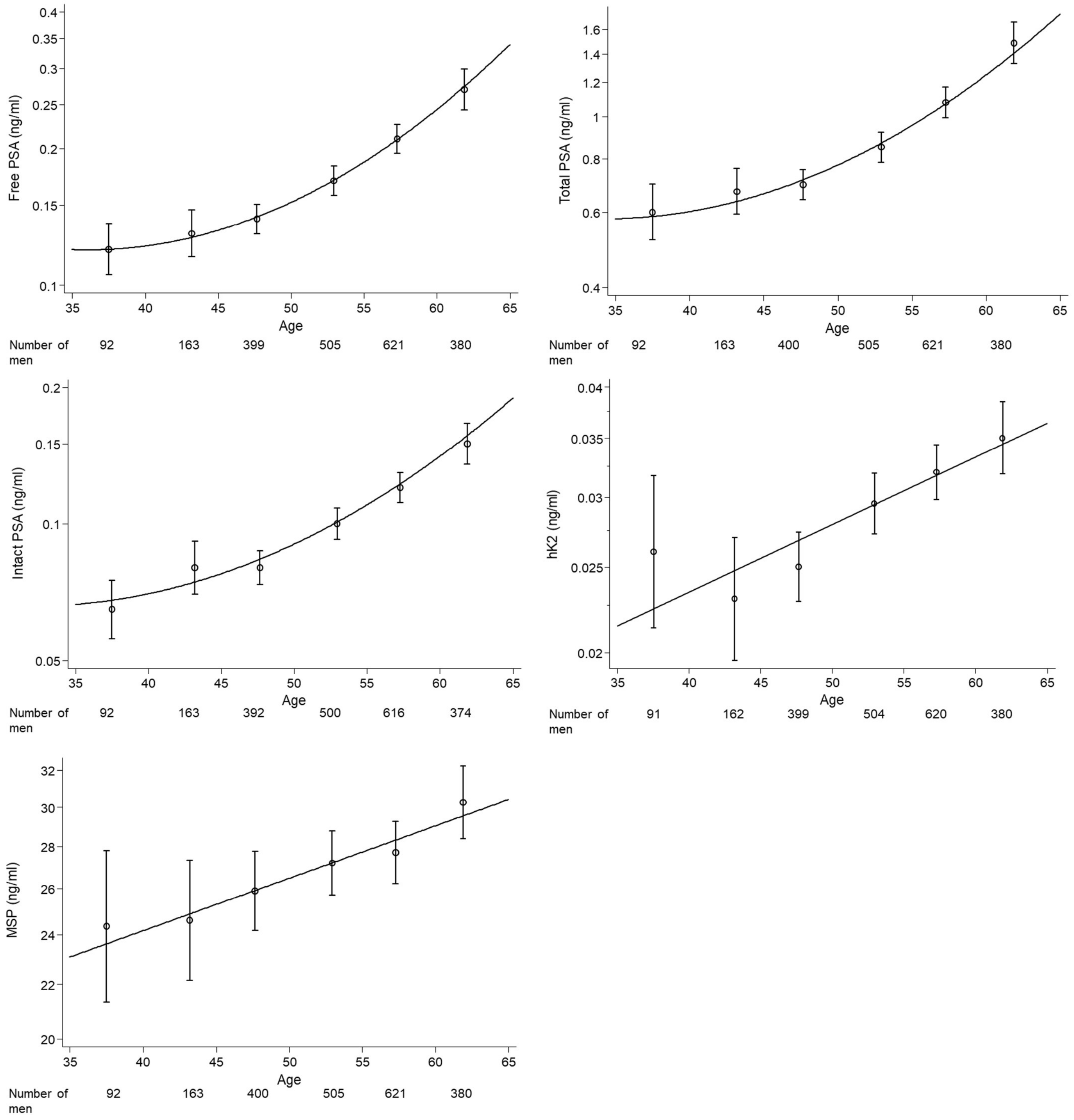

$\log_{10}$(Free PSA) = $-0.22124461-0.0388905\times age+0.0005393\times age^2$; $\log_{10}$(Total PSA) = $0.3506266-0.0344012\times age+0.0005028\times age^2$; $\log_{10}$(Intact PSA) = $-0.7524686-0.02684\times age+0.0004198\times age^2$; $\log_{10}$(hK2)=$-1.937678+0.0076657\times age$; $\log_{10}$(MSP)=$1.22319+0.0039945\times age$

Figure S2: Probability plots of markers in affected (died of, or with a history of prostate cancer) and unaffected men with up to and including 5 years of follow-up

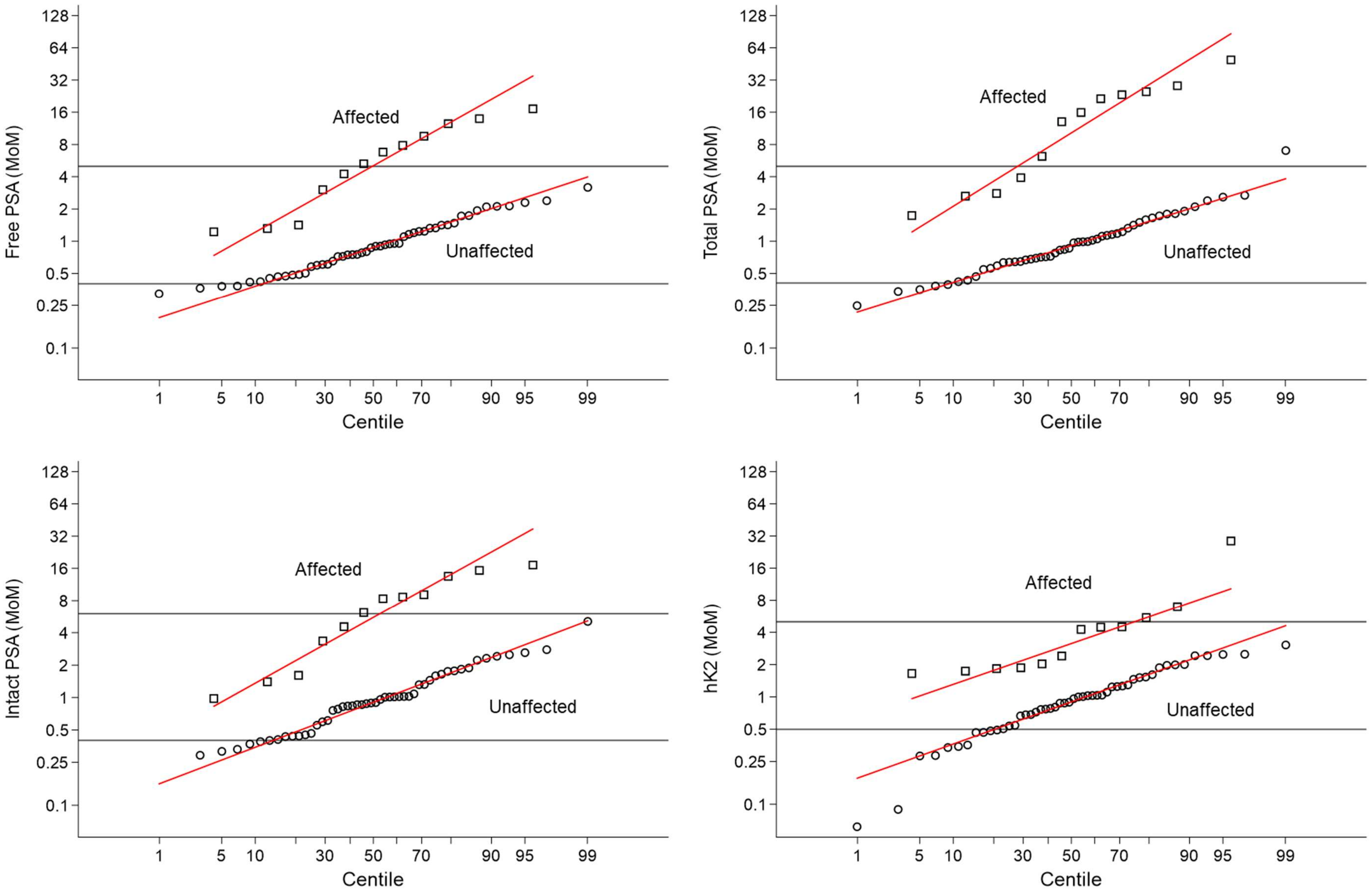

Figure S3: Probability plots of markers in affected (died of, or with a history of prostate cancer) and unaffected men with up to 10 years of follow-up

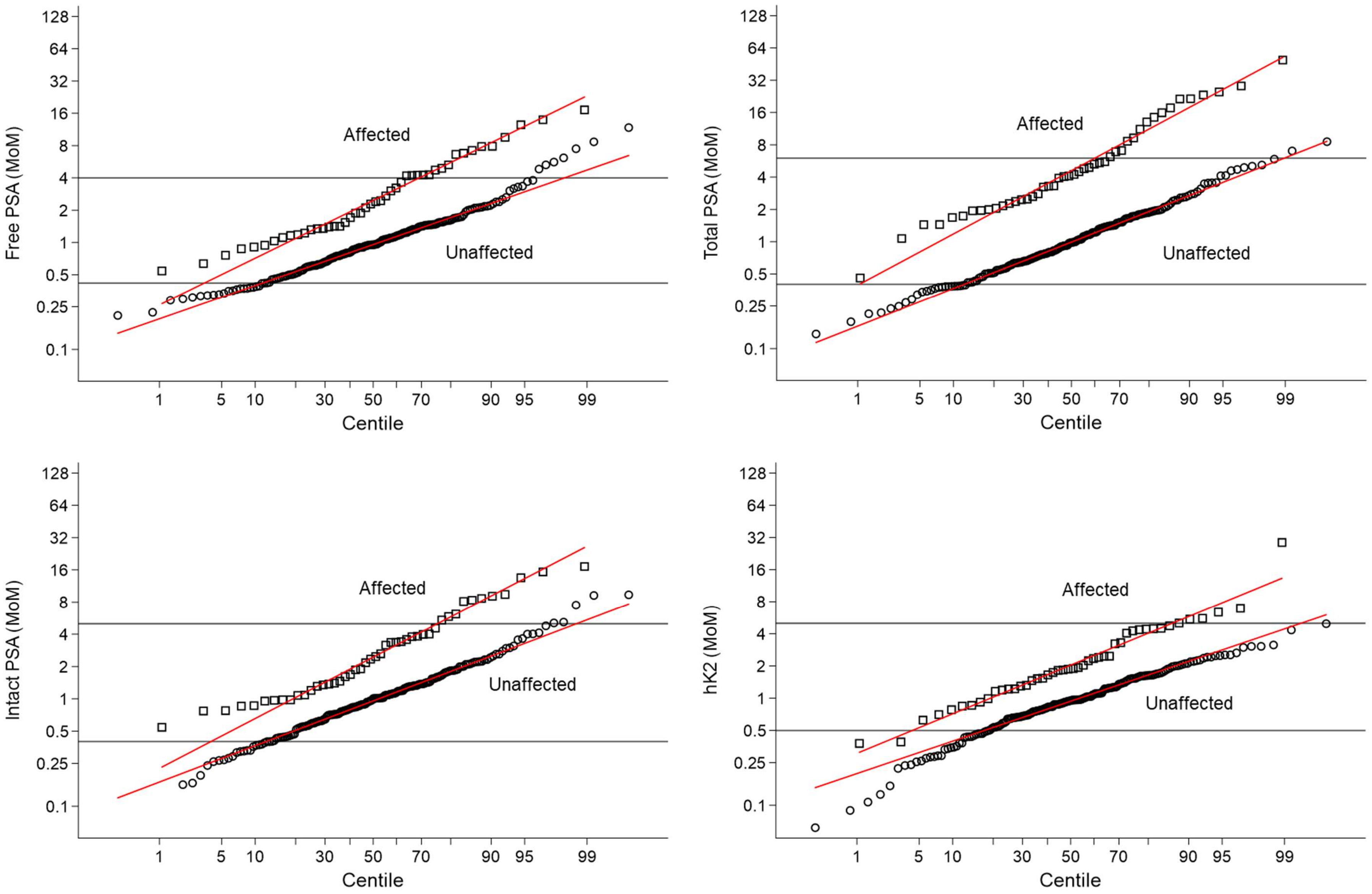

Figure S4: Median marker MoM values (and 95% confidence intervals) in unaffected men according to body mass index (BMI) (<20, 20-22.5, 22.5-25, 25-27.5, 27.5-30, ≥30), together with regression lines

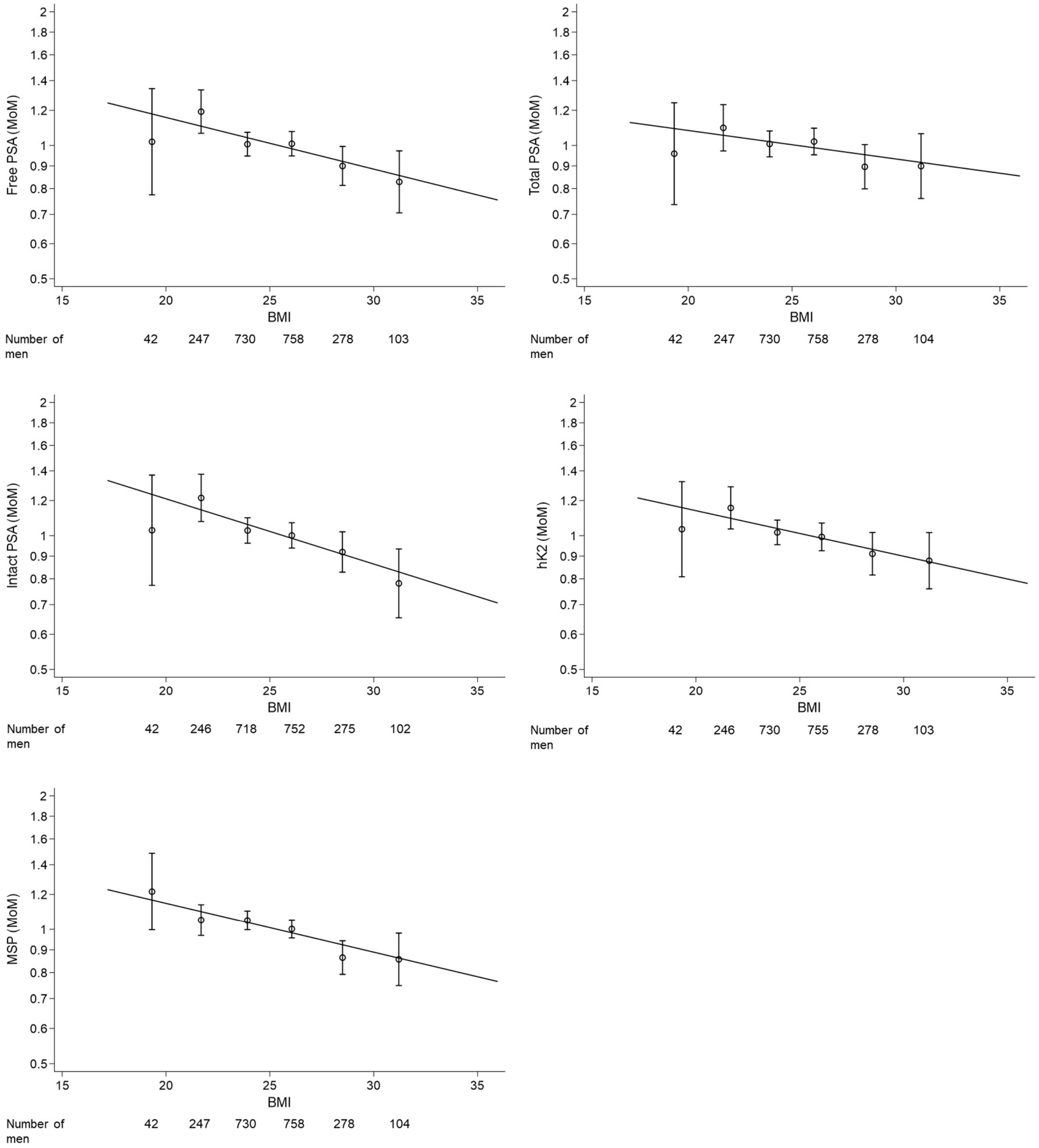

$\log_{10}$(Free PSA MoM)=0.2956844-0.0116291 ×BMI; $\log_{10}$(Total PSA MoM)=0.1619973-0.006411×BMI; $\log_{10}$(Intact PSA MoM)=0.376408-0.0146729×BMI; $\log_{10}$(hK2 MoM)=0.2611946-0.0102456×BMI; $\log_{10}$(MSP MoM)=0.2791327-0.0110107×BMI.
